# Exercise Training Prevents the Loss of Wall Thickness and Lowers Expression of Alzheimer’s Related Proteins in 3xTg Mouse Jejunum

**DOI:** 10.3390/ijerph192114164

**Published:** 2022-10-29

**Authors:** Layla Al-Nakkash, Daniel Mason, Niamatullah Ismail, Taylor Bowman, John Ahlert, Maxwell Rubin, Emma Smith, Abigail Rosander, Tom L. Broderick

**Affiliations:** 1 Arizona College of Osteopathic Medicine, Midwestern University, Glendale, AZ 85308, USA; 2 Department of Physiology, College of Graduate Studies, Midwestern University, Glendale, AZ 85308, USA; 3 College of Veterinary Medicine, Midwestern University, Glendale, AZ 85308, USA

**Keywords:** jejunum, exercise, 3xTg, alzheimer’s disease

## Abstract

Growing evidence has demonstrated the benefits of regular exercise on cardiovascular, neural, and cognitive function in humans with Alzheimer’s disease (AD). However, the consequences of AD on gastrointestinal morphology and the effects of regular exercise, which plays an important role against the development of certain gastrointestinal-related diseases, are still poorly understood. Therefore, to assess the changes in intestinal structure in a mouse model of AD and the impact of exercise, 2-month-old 3xTg-AD male mice were subjected to treadmill running 5 days per week for a period of 5 months. Jejunum from 3xTg-AD mice analyzed by histochemical methods revealed significant alterations in morphology. Compared to age-matched wild-type (WT) mice, villi length and crypt depth were increased, and collagen content of jejunum was elevated in 3xTg-AD mice. Jejunum wall dimensions, expressed as total wall thickness, outer longitudinal thickness, and inner circular thickness were decreased in 3xTg-AD compared to WT. Smooth muscle actin expression in jejunal wall was decreased in 3xTg-AD. Most of these aberrations were improved with exercise. Western blot expression of cyclin dependent kinase 5 (CDK5, involved in neural cell death and hyperphosphorylation of tau), was elevated in 3xTg-AD jejunum. This was associated with a 4-fold increase in tau5 expression. Exercise prevented the increase in expression of CDK5 and tau5. Expression of caspase 3 (an apoptotic marker) was elevated in 3xTg-AD jejunum and exercise prevented this. The results of our study indicate that the abnormalities in jejunum of the 3xTg mouse model of AD were prevented with exercise training.

## 1. Introduction

The role of habitual aerobic exercise on gastrointestinal (GI) function and health is an area of interest. While exercise may provoke gastrointestinal symptoms such as heartburn, nausea, vomiting, and diarrhea, these unwanted consequences are typically reported with prolonged and intense exercise [1]. On the other hand, exercise of mild-to-moderate intensity, is well-tolerated and generally beneficial to GI health. In support of this, several studies have demonstrated an inverse relationship between regular physical activity and the risk of developing GI and liver-related diseases, such as diverticular disease, inflammatory bowel disease, and cholelithiasis [2,3,4]. Thus, an exercise recommendation to prevent undesirable GI effects while promoting benefits on GI health includes a reduction in exercise intensity which not only is appropriate for the aging population, but also known to improve health outcomes in this population that may also be afflicted by other chronic age-related diseases [5].

Alzheimer’s disease (AD) is a debilitating neurodegenerative disorder associated with impairment in cognitive function, accounting for most cases of dementia [6]. The neuropathological hallmarks of AD include the accumulation of beta-amyloid plaques and intracellular tau tangles in the brain [7,8]. Although the pathogenesis has been extensively characterized within the brain, beta-amyloid plaque deposition has been detected in other tissues from AD patients, including heart and intestine [9,10,11,12]. While AD is primarily a disease of the brain and manifests as the well-known debilitating symptoms over time, there is also increasing documentation to demonstrate the negative influence of AD and/or aging on other organs including the gastrointestinal system. For example, dysfunctions in the autonomic nervous system can cause constipation, noted in patients with AD [13,14,15,16,17]. Murine models of AD mimic some of the intestinal aberrations seen clinically, i.e., decreased intestinal motility and constipation [18,19,20]. The effect of AD on gastrointestinal health is still a relatively poorly understood area. To date, current therapy exists only for the relief of symptoms and no effective treatment is available despite the efforts in the development of therapeutic drugs. Therefore, alternative approaches to prevent or delay the onset of AD need to be explored. One approach, which is safe and effective in delaying the development of neurodegeneration in AD, is regular exercise.

A wealth of studies indicate that regular exercise and leisure-time physical activity affords significant neuroprotection in humans and murine models of AD, thus slowing the progression of this disease [21,22,23,24,25]. We have recently demonstrated that exercise also offers physiological resilience and protection in other tissues, including bone, heart, and aorta. Chronic moderate intensity exercise improved tibial bone quality and increased fracture resistance in the 3xTg-AD mouse model [26]. Moreover, the abnormal indices of cardiac systolic and diastolic function and the disrupted architecture of elastin within smooth muscle layer of aorta were prevented after exercise training [27].

Based on these observations, it is possible that the practice of exercise may result in important changes in the GI tract that might benefit patients with AD. Exercise may be an approach to alleviate the GI effects of AD which includes dysautonomia, reduced gut transit time, and constipation [28]. In this study, using the 3xTg-AD mouse, we investigated the effects of chronic moderate intensity aerobic exercise in the form of treadmill running on basic morphology of the small intestine and on the expression of certain proteins linked to synaptic neuronal death and neural dysfunction.

## 2. Materials and Methods

### 2.1. Mouse Model and Exercise Protocol

The Midwestern University Institutional Animal Care and Use Committee approved all experiment protocols as described in this study. Male triple transgenic mice aged 8-weeks (3xTg-AD, Jackson Laboratories, Bar Harbor, ME, USA), were used as a model for AD. This model harbors three mutant genes (Aβ precursor protein, presenilin-1, and tau) and exhibits parallels to human AD [29,30]. The 3xTg-AD mice were compared to a control wild-type AD free group (age-matched, non-transgenic wild type mice, B6129SS2/J). Male mice were utilized because of prior evidence of running without reluctance [31] and because men have a shorter lifespan after the diagnosis of AD, compared to women [32,33].

**Figure 1:**
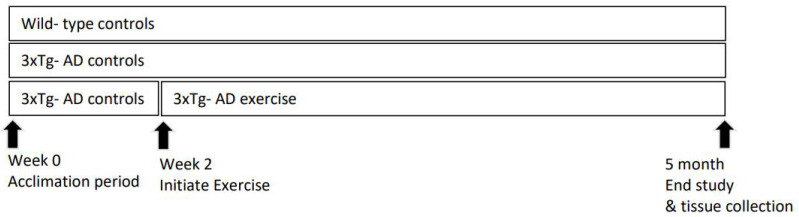
A schematic demonstrating the study timeline.

Mice were assigned one of the three following groups: wild-type control (WT), 3xTg-AD control (AD), 3xTg-AD mice exercise trained (AD + Ex). Exercise training was accomplished using an electric treadmill (Exer 3/6, Columbus Instruments, Columbus, OH, USA) as described previously [31]. Briefly, mice were acclimated to daily 10 min exercise sessions at 10 m/min for an acclimation period of two-weeks. After the acclimation period, training commenced and consisted of a graded increase in duration and intensity until mice performed 45 min sessions at 15 m/min (week 1 = 20 min at 10 m/min, week 2 = 30 min at 12 m/min, weeks 3–5 month = 45 min at 15 m/min). Mice in the exercised groups ran with ease and completed the exercise protocol for the 5-month duration of the study. A schematic of the study timeline is shown in Figure 1. No signs of poor health, distress or differences in body weight and food intake between groups of mice were observed during the exercise period [27]. Mice were housed two per cage and maintained in a room with a 12:12 h light–dark cycle and given food and water ad libitum. Following exercise training, mice were euthanized by CO_2_ gas followed by bilateral pneumothorax. Tissues were harvested, and immediately frozen in liquid nitrogen and stored at −80 °C until use.

### 2.2. Histology and Morphology

Segments of mouse jejunum were embedded, and frozen in Optimal Cutting Temperature compound (O.C.T., Tissue-Tek, Torrance, CA, USA). Hematoxylin and eosin (H & E) staining was performed as previously described [34,35]. Crypt depth, villi length, total wall thickness, outer longitudinal thickness and inner circular thickness were measured on images, using Image J (NIH). Trichrome staining was performed according to previously described methods [36]. Trichrome staining was analyzed using Halo software (Indica Labs, Albuquerque, NM, USA). The slides were individually annotated, and stain area quantification performed. Digital representations of stains were chosen on a RBG scale from an average sample of positive stain pixels. Absolute areas of stain and percent of stain were quantified. To calculate percent area tissue, empty space was excluded from the analysis and the percentage of stain is the quotient of positive stain over the total actual tissue area.

### 2.3. Western Blot Analysis

Frozen segments of jejunum were prepared for Western blot assessments using similar Western blot methodology as previously described [35,37,38]. Protein Assays were performed to determine homogenate concentration. Blots were incubated with the following primary antibodies overnight at 4 °C: SGLT-1 (1:500, Abcam Cambridge, UK), GLUT2 (1:500, Cell Signaling, Danvers, MA, USA), GLUT5 (1:3000, Cell Signaling, Danvers, MA, USA), Tau 5 (1:750, Calbiochem, Millipore-Sigma, Burlington, MA, USA), CDK5 (1:750, Cell Signaling, Danvers, MA, USA), Caspase-3 (1:1000, Cell Signaling, Danvers, MA, USA), Alpha actin smooth muscle (1:750, Invitrogen, Thermo-Fisher, Waltham, MA, USA). To re-probe for GAPDH, blots were incubated with anti-GAPDH primary antibody (1:4000, Thermo Scientific, Rockford, IL, USA) or anti-actin (1:3000, EMD Millipore, Billerica, MA, USA) for 1 h at room temperature. Blots were washed and then incubated with the appropriate secondary antibody anti-mouse IgG (H + L) (1:15,000, Dylight, Thermo Scientific, Rockford, IL, USA), and anti-rabbit IgG (H + L) Dylight (1:15,000, Thermo Scientific, Rockford, IL, USA), for 1 h at room temperature. Images of membranes were taken with all proteins of interest normalized to either actin or GAPDH. Band density was analyzed using Odyssey-Clx (LI-COR, Lincoln, NE, USA) and Image Studio (LI-COR, Lincoln, NE, USA).

### 2.4. Statistical Analysis

Data are expressed as mean ± SEM. One-way ANOVA with Dunnett’s multiple comparison test was performed using GraphPad (San Diego, CA, USA). Numbers in parentheses represent numbers of tissues used from separate individual mice. *p* < 0.05 was considered statistically significant.

## 3. Results

### 3.1. The Effects of Exercise Training on Food Intake and Phenotype of 3xTg-AD Mice

As described previously, there were no differences in either body weight or food intake in the three groups of mice [27,31]. Body weights were: WT 39.2 ± 2.4 (n = 10), 3xTg-AD 34.9 ± 1.6 (n = 7), and 3xTg-AD with exercise 35.6 ± 1.1 (n = 8). The absence of effects could be attributed to potential influences on the metabolism of the 3xTg-AD at this age (~seven months of age). Indeed, age-related changes in food intake and body weight of triple-transgenic mice have been assessed by others and shown to be related to changes in metabolic state [39].

### 3.2. The Effects of Exercise Training on Jejunal Morphology of 3xTg-AD Mice

**Figure 2:**
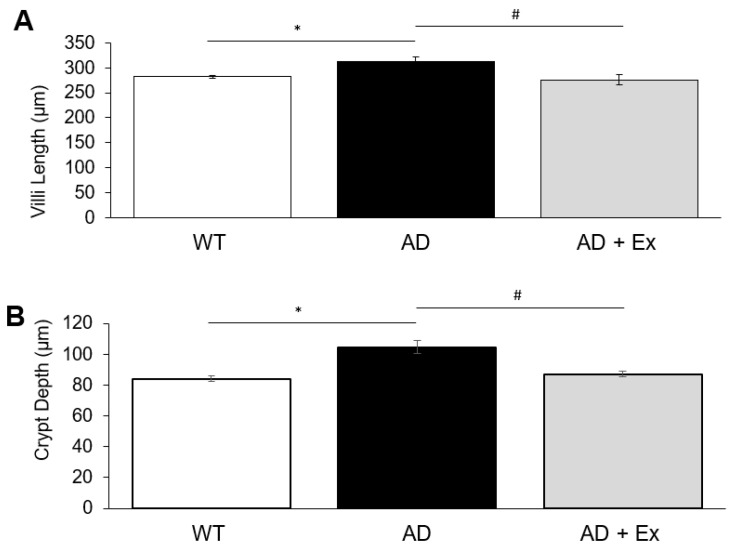
The effects of exercise training on villi dimensions in 3xTg-AD mouse jejunum. Villi length (**A**) and villi depth (**B**), in μM, of jejunum from 3xTg-AD mice. Values are reported as mean ± SEM for 7–8 mice per group. * indicates significance compared to WT control mice, *p* < 0.05; # indicates significance compared to 3xTg-AD mice, *p* < 0.05. AD, Alzheimer’s disease; WT, wild type; Ex, exercise.

Analysis of jejunum isolated from mice, and stained with hematoxylin and eosin, to determine tissue morphology, revealed significant changes relating to this model of AD and exercise training. As illustrated in Figure 2, both villi length and crypt depth were altered in jejunum of 3xTg-AD mice and by exercise. Villi length and crypt depth were increased 1.1- and 1.24-fold, respectively, in 3xTg-AD mice compared to WT control mice, with exercise maintaining length and depth at control values.

**Figure 3:**
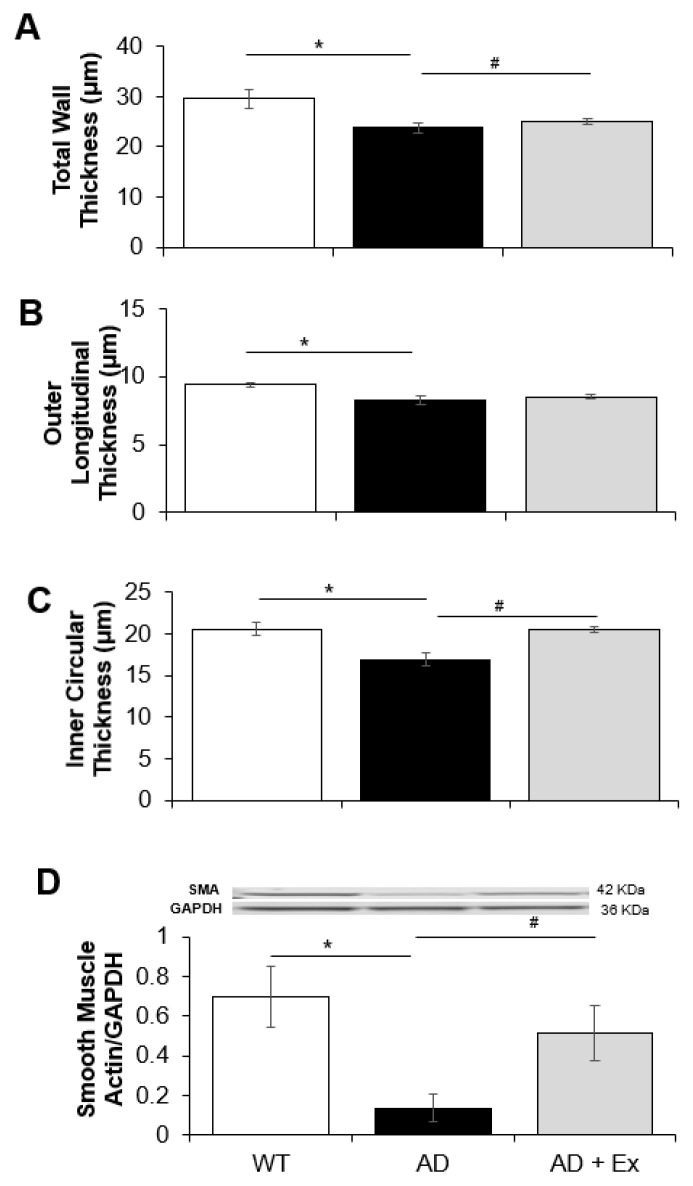
The effects of exercise training on wall dimensions and smooth muscle actin expression in 3xTg-AD mouse jejunum. Total wall thickness (**A**), outer longitudinal wall thickness (**B**), inner circular wall thickness (**C**) and total protein expression of smooth muscle actin expression (**D**) of jejunum from 3xTg-AD mice. Wall thicknesses are expressed in μM. Protein expression of smooth muscle actin was normalized using GAPDH. Values are reported as mean ± SEM for 7–9 mice per group for the wall dimensions and 5–8 mice per group for smooth muscle actin. * indicates significance compared to WT control mice, *p* < 0.05; # indicates significance compared to 3xTg-AD mice, *p* < 0.05. AD, Alzheimer’s disease; WT, wild type; Ex, exercise.

Jejunal wall dimensions were also altered in 3xTg-AD and exercise training. As shown in Figure 2, there was a significant 20% decrease in total wall thickness in the 3xTg-AD mice compared to WT control mice (Figure 3A). The decrease in total wall thickness was associated with concomitant changes in both outer longitudinal smooth muscle and inner circular smooth muscle layers (Figure 3B,C). Exercise training increased the inner wall thickness only resulting in a significant increase in total wall thickness of the jejunum (Figure 3C). The noted changes in wall thickness were associated with a similar effect on total protein expression of smooth muscle actin in jejunum. Expression of smooth muscle actin in the 3xTg-AD mice was significantly lower (0.13 ± 0.07, n = 5, *p* < 0.05), compared to WT mice (0.69 ± 0.16, n = 7, *p* < 0.05). Exercise training improved the expression of smooth muscle actin, resulting in increased levels (0.51 ± 0.14, n = 8, *p* < 0.05) comparable to that observed in WT control mice (Figure 3D).

**Figure 4:**
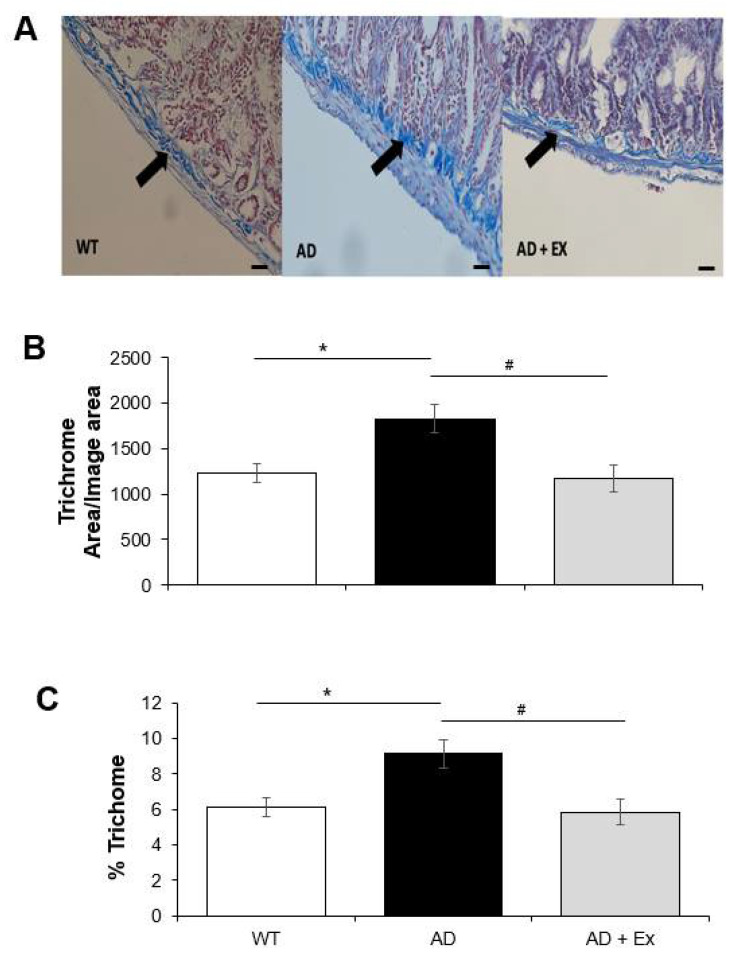
The effects of effects of exercise training on collagen content in 3xTg-AD mouse jejunum. Trichrome staining was used to determine collagen content. Representative 20× images are shown (**A**), trichrome area (representing the ratio of trichrome stained area over the entire area evaluated, and expressed as arbitrary units) (**B**) and percentage of trichome (**C**). Scale bars are 50 µm. Values are reported as mean ± SEM for 23–31 measurements per group. * indicates significance compared to WT control mice, *p* < 0.05; # indicates significance compared to 3xTg-AD mice, *p* < 0.05. AD, Alzheimer’s disease; WT, wild type; Ex, exercise.

Collagen fibers are of biomechanical importance in any portion of the GI tract as they allow for longitudinal distension while limiting excessive expansion. Increased collagen in the GI tract is linked to intestinal stiffness [40]. To further assess changes in intestinal morphology of 3xTg-AD mice, we measured collagen deposition in sections of jejunum using total trichrome staining. As shown in Figure 4, trichrome staining for collagen content, expressed as total area or percentage, was increased ~1.5-fold in 3xTg-AD mice compared to control WT mice. Exercise training was effective in reducing the amount of collagen content in jejunum of 3xTg-AD mice to control (WT) levels.

### 3.3. The Effects of Exercise Training on Expression of Absorptive Proteins in Jejunum of 3xTg-AD Mice

**Figure 5:**
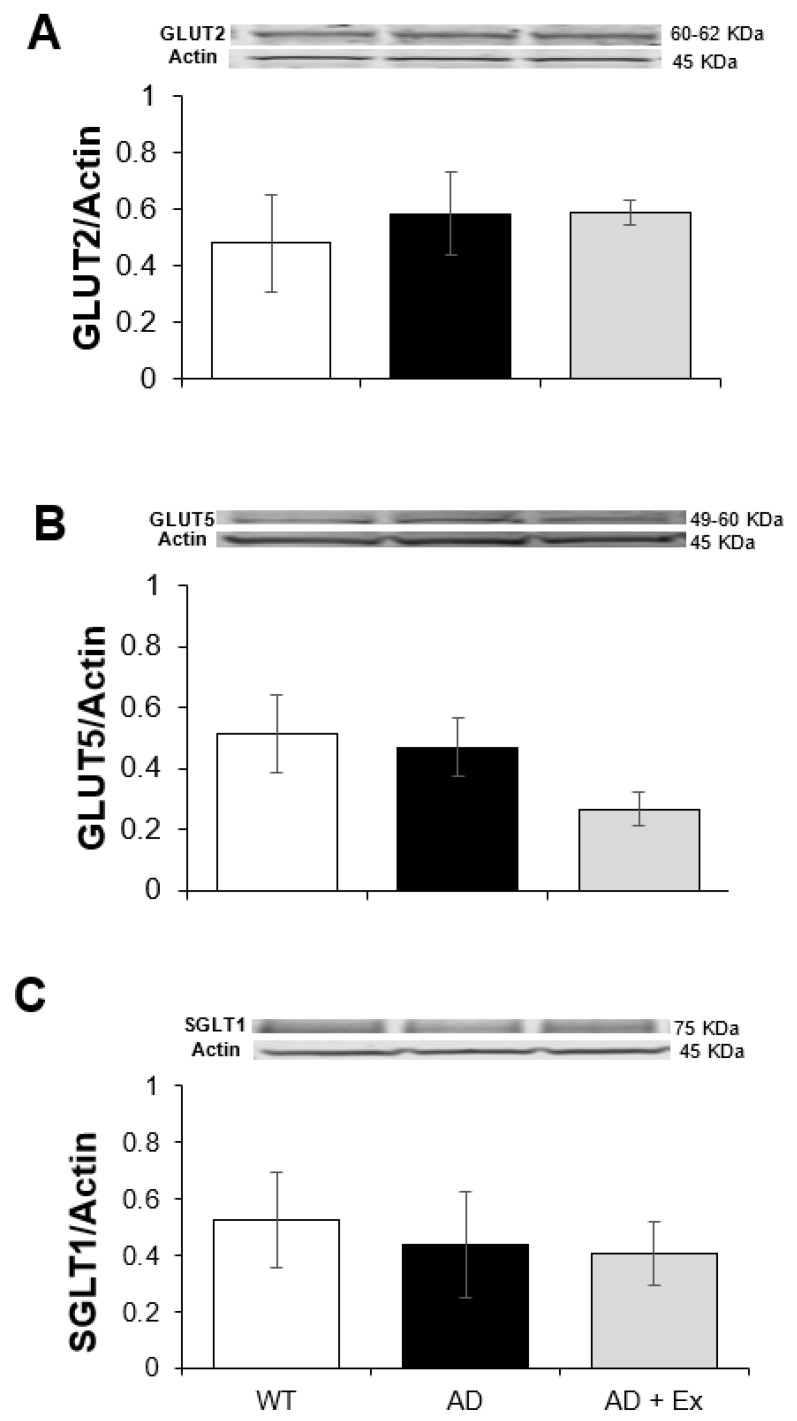
The effects of exercise training on protein expression of the absorptive intestinal transporters in 3xTg-AD mouse jejunum. GLUT2 (**A**), GLUT5 (**B**), and SGLT-1 (**C**) from jejunum of 3xTg-AD mice. Values are reported as mean ± SEM for 6–8 mice per group. GLUT2/GLUT5, glucose transporter; SGLT-1, sodium-glucose cotransporter; AD, Alzheimer’s disease, WT, wild type; Ex, exercise.

Mice were sacrificed at the age of 7 months, corresponding to an age when potential modifications in intestinal proteins responsible for the absorption of monosaccharide products of digestion occur. Therefore, we measured the total expression of monosaccharide transporters 2 and 5 (GLUT2, GLUT5) and sodium-glucose cotransporter (SGLT-1). Fructose uptake occurs via GLUT5 on the apical membrane, SGLT1 facilitates the uptake of glucose/galactose on the apical membrane, and GLUT2 mediates fructose/glucose/galactose on the basolateral membrane across jejunum epithelia. Expression of these transport proteins in jejunum is illustrated in Figure 4. There were no significant differences in the expression levels of GLUT2 (Figure 5A), GLUT5 (Figure 5B) and SGLT1 (Figure 5C) between WT mice, 3xTg-AD mice, and 3xTg-AD mice subjected to 5 months of exercise training. These data suggest that a 10% modest change in villi length in the 3xTg-AD mice was not associated with concomitant changes in the transporters measured.

### 3.4. The Effects of Exercise Training on Expression of AD-Related Proteins in Jejunum of 3xTg-AD Mice

**Figure 6:**
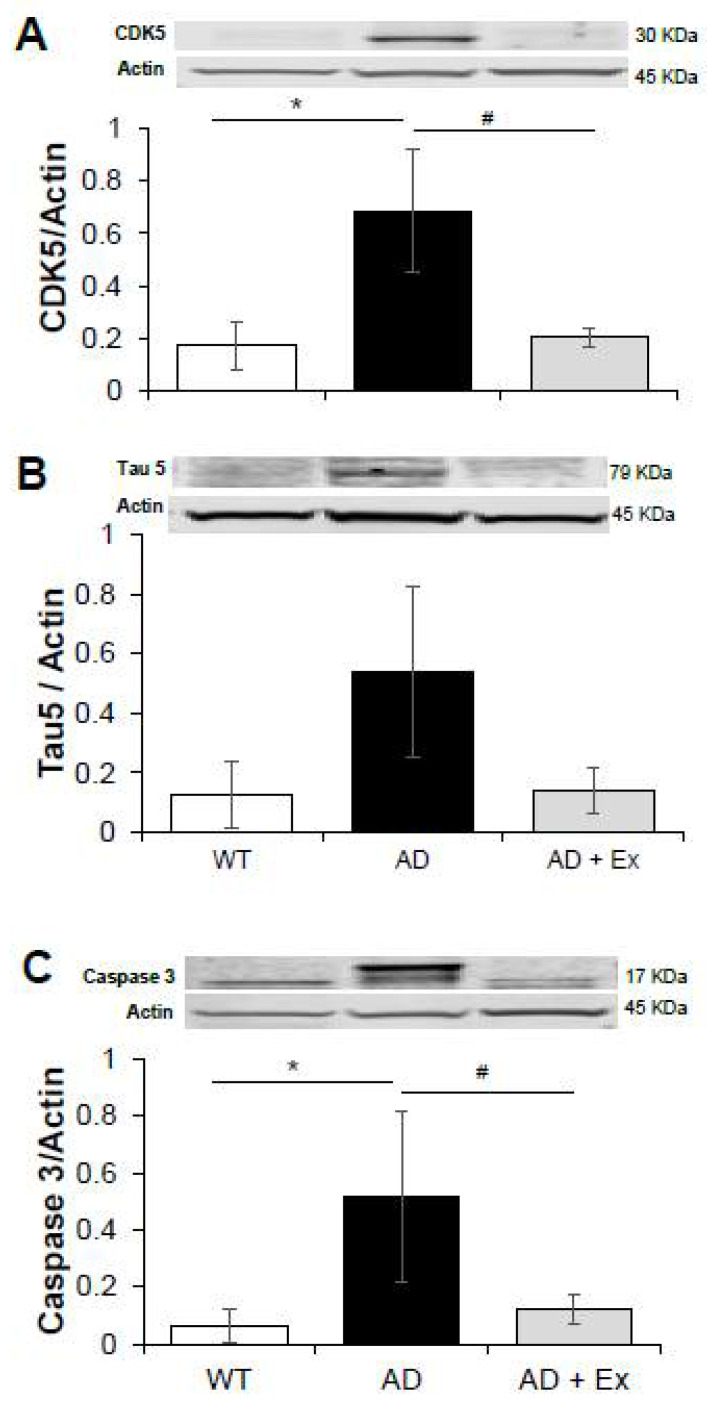
The effects of exercise training on total protein expression of AD-related proteins in 3xTg mouse jejunum. CDK5 (**A**), Tau5 (**B**) and caspase 3 (**C**) of jejunum from 3xTg-AD mice. Values are reported as mean ± SEM for 3 to 5 mice per group. * indicates significance compared to WT control mice, *p* < 0.05; # indicates significance compared to 3xTg-AD mice, *p* < 0.05. CDK, cyclin dependent kinase; AD, Alzheimer’s disease; WT, wild type; Ex, exercise.

Cyclin dependent kinase 5 (CDK5) is a serine threonine kinase that exerts a vital role in the development of the central nervous system, synaptic plasticity, and microtubule regulation [41]. Dysregulation of CDK5 has been implicated in synaptic dysfunction and neuronal death by producing the aberrantly phosphorylated tau in AD, contributing to neural plaques and neurofibrillary tangles [42,43,44]. As shown in Figure 6A, expression of CDK5 was significantly increased in jejunum of 3xTg-AD mice compared to WT control mice (0.69 ± 0.23 vs. 0.17 ± 0.09, *p* < 0.05). Exercise training was beneficial and prevented the increase in CDK5 (0.20 ± 0.04, *p* < 0.05). Since dysregulation of CDK5 leads to hyperphosphorylation of its physiological target Tau, we then measured Tau5 expression in jejunum. Compared to WT mice, expression of Tau5 was increased almost 4-fold in 3xTg-AD mice, although this was not statistically significant (Figure 6B). Protein levels of Tau5 in 3xTg-AD mice were decreased to control levels after exercise training, but again this effect was not significant.

Caspase 3 is an enzyme marker involved in the apoptotic execution pathway of tissues. Caspase 3 has been shown to be activated with caspases 8 and 9, together leading to apoptotic processing of the cell [45]. To determine whether the decreased jejunum wall thickness in 3xTg-AD mice was caused by apoptosis, total caspase 3 protein levels were determined (Figure 6C). We found that expression of caspase 3 in jejunum of 3xTg-AD mice was significantly increased compared to WT mice (0.51 ± 0.29 vs. 0.06 ± 0.05, respectively, *p* < 0.05). Exercise training prevented the age-related change in caspase-3 expression (0.11 ± 0.05, *p* < 0.05), which could explain the exercise-induced improvement in wall thickness (Figure 3A,C).

In summary, our data show that this mouse model of AD is associated with changes in jejunum wall morphology. In 7-month-old male 3xTg-AD mice, alterations in villi length and crypt depth, jejunum wall thickness, collagen content, and protein levels of smooth muscle active, CDK5 and caspase 3 were observed. A period of 5 months of regular treadmill running had beneficial effects on this tissue.

## 4. Discussion

An emerging theme in studies examining the role of non-pharmacological therapy in the treatment of AD is the effectiveness of habitual exercise in delaying the progression of abnormal neuropathology, resulting in improved quality of life in patients. The beneficial effects of chronic exercise training on brain neurodegeneration in humans and murine models of AD are well-documented. However, there is an increasing number of studies supporting the claim that exercise also favorably prevents the AD-related decline of other physiological systems, including those of cardiovascular and cerebrovascular [46], cardiorespiratory [47], and musculoskeletal [48,49] systems.

Using the 3xTg mouse to study the impact of chronic exercise on system responses in AD, our recent work has shown that regular exercise training not only prevented neurodegeneration, but also improved cardiovascular function and increased bone fracture resistance [26,27,31]. In this current study, we extend these observations by providing evidence that habitual aerobic exercise has beneficial effects on intestinal tissue obtained from the 3xTg-AD mouse. These effects consisted mainly of reversing the defects in jejunum villi and wall morphology and decreasing collagen content. In addition, exercise training exerted a positive effect on CDK5 and caspase 3 expression in jejunum, proteins known to be triggered in brain and neural tissue and implicated in the pathogenesis of neurodegeneration [41,50].

A widened view of AD as a peripheral disease involving dysautonomia permeating several organs is now evolving and receiving attention. Disturbances in the autonomic nervous system result in dysfunctions such as constipation, which is also observed in patients with AD [13,14,15,16,17]. Relevant experimental models, including female APP^NL-G-F^, male and female Tg2576, and male 5xFAD murine models of AD, also exhibit similar bowel dysfunction such as decreased intestinal motility and constipation [18,19,20]. The pathogenesis of constipation in AD is not entirely clear, but recent evidence suggests that morphological changes and damage within the GI tract may be responsible. Constipation observed in 11-month-old T2576 mice with deficient cholinergic signaling was associated with histopathological changes, including abnormal crypt depth, and decreased thickness of mucosal and muscle layers [19]. We report similar morphological changes, albeit in a different model of AD and in much younger mice. GI dysautonomia using physiological assessments was not confirmed in the current study. We also show an increase in total caspase 3 expression, suggesting that cell damage may explain the decreased longitudinal and circular wall thickness thereby altering the architecture of the intestinal wall. Given these observations, it is evident that the pathology in 3xTg-AD mice correlates with adverse effects on GI morphology and function.

Implementation of regular exercise in 3xTg-AD mice produced beneficial effects on gut morphology. Exercise produced favorable changes in villi and crypt dimensions, increased jejunal longitudinal and circular wall thickness, and decreased collagen content. These changes are likely indicative of improved GI health in the 3xTg model of AD. In addition, expression of caspase 3 was lower in jejunum of exercising 3xTg-AD mice, suggesting reduced damage and preserved jejunum wall integrity. Given the importance of the longitudinal and circular muscle layers in intestinal peristalsis, these effects could be interpreted as an additional beneficial outcome of exercise. In fact, this is consistent with the view that maintenance of circular wall integrity enhances contractile function of ileum whereas hypertrophy of the longitudinal layer is associated with increased responsiveness to relaxing factors [51,52]. Furthermore, a reduction in collagen within the jejunum of exercising mice suggests decreased intestinal stiffness, likely improving intestinal motility [40]. Cognitive deficiencies in AD are thought to be associated with changes in the gut microbiome, and importantly, exercise has been shown to alter the composition of the microbiome in AD, resulting in improved cognitive function and memory [53]. Whether or not exercise-induced or was associated with changes in the microbiome of the 3xTg-AD mice in this study remains to be seen. However, it is possible that exercise-induced benefits to overall microbial composition convey modifications to intestinal inflammation and GI structure/protein expression in the 3xTg-AD mice. Future studies will aim to address such interactions.

Dysregulation of CDK5 is implicated in the formation of beta-amyloid and neurofibrillary tangles, both abnormal neurological hallmarks of AD [41]. With evidence that these hallmark proteins may spread via anatomical connectivity [54], the effects of hyperactivation of CDK5 are suggested to contribute to neuronal loss of the enteric nervous system in ileum of APP/PSa AD mice and human AD colon [55,56,57]. We observed that CDK5 expression was elevated in jejunum of 3xTg-AD mice. Since dysregulation of CDK5 is involved in neurodegeneration and synaptic dysfunction, therefore, increased expression levels in jejunal wall may suggest an impact on intestinal motility potentially from loss of enteric function [55,58]. The increase in jejunum CDK5 expression was prevented with chronic exercise training. Similarly, a previous study demonstrated a decrease in CDK5 hyperactivation in hippocampus of gp120 transgenic mice, a model of impaired neurogenesis, following 20 days of voluntary wheel running [59], which resulted in significant improvements in neurogenesis and normalized brain-derived neurotrophic factor levels.

In conclusion, the results of this study show significant morphological alterations in jejunum and proteins associated with neural damage of the GI tract of 3xTg-AD mice. In addition, this study demonstrated that exercise training for 5 months, resulted in notable beneficial effects, alleviating the abnormal changes observed in the jejunum of 3xTg-AD mice. This gastroprotective effect of exercise highlights the role of this non-pharmacological therapy as a means to potentially promote or delay AD-related pathology.

## Data Availability

Data are available upon request to the corresponding author.

## References

[1] Peters H.P., Bos M., Seebregts L., Akkermans L.M., van Berge Henegouwen G.P., Bol E., Mosterd W.L., de Vries W.R. (1999). Gastrointestinal symptoms in long-distance runners, cyclists, and triathletes: Prevalence, medication, and etiology. Am. J. Gastroenterol..

[2] Colditz G.A., Cannuscio C.C., Frazier A.L. (1997). Physical activity and reduced risk of colon cancer: Implications for prevention. Cancer Causes Control.

[3] Aldoori W.H., Giovannucci E.L., Rimm E.B., Ascherio A., Stamfer M.J., Colditz G.A., Wing A.L., Trichopoulos D.V., Willett W.C. (1995). Prospective study of physical activity and the risk of symptomatic diverticular disease in men. Gut.

[4] Leitzmann M.F., Giovannucci E.L., Rimm E.B., Stampfer M.J., Spiegelman D., Wing A.L., Willett W.C. (1998). The relation of physical activity to risk for stmptomatic gallstone disease in men. Ann. Intern. Med..

[5] Peters H.P.F., De Vries W.R., Vanberge-Henegouwen G.P., Akkermans L.M.A. (2001). Potential benefits and hazards of physical activity and exercise on the gastrointestinal tract. Gut.

[6] Scheltens P., De Strooper B., Kivipelto M., Holstege H., Chetelat G., Teunissen C.E., Cummings J., van der Flier W. (2021). Alzheimer’s Disease. Lancet.

[7] Hardy J., Selkoe D.J. (2002). The amyloid hypothesis of Alzheimer’s disease: Progress and problems on the road to therapuetics. Science.

[8] Haas C., Selkoe D.J. (2007). Suluble protein oligomers in neurodegeneration: Lessons from the Alzheimer’s amyloid beta-peptide. Nat. Rev. Mol. Cell. Biol..

[9] Joachim C.L., Mori H., Selkoe D.J. (1989). Amyloid beta-proetin deposition in tissues other than brain in Alzheimer’s disease. Nature.

[10] Tronchone L., Luciani M., Coggins M., Wilker E.H., Ho C.-Y., Codispoti K.E., Frosch M.P., Kayed R., Del Monte F. (2016). AB Amyloid pathology affects the hearts and minds of patients with Alzheimer’s disease: Mind the heart. J. Am. Coll. Cardiol..

[11] Dugger B.N., Whiteside C.M., Maarouf C.L., Walker D.G., Beach T.G., Sue L.I., Garcia A., Dunckley T., Meechoovet B., Reinman E.M. (2016). The presence of select tau species in human peripheral tissues and thier relation to Alzheimer’s disease. J. Alzheimers Dis..

[12] Honarpisheh P., Reynolds C.R., Blasco Conesa M.P., Manchon J.F.M., Putluri N., Bhattacharjee M.B., Urayama A., McCullough L.D., Ganesh B.P. (2020). Dysregulated gut homeostasis observed prior to the accumulation of the brain amyloid-B in Tg2576 mice. Int. J. Mol. Sci..

[13] Wang S.J., Liao K.K., Fuh J.L., Lin K.N., Wu Z.A., Liu C.Y., Liu H.C. (1994). Cardiovascular disease autonomic functions in AlzHeimer’s disease. Age Ageing.

[14] Szili-Torok T., Kalman J., Paprika D., Dibo G., Rozsa Z., Rudas L. (2001). Depressed baroreceptor reflex sensitivity in patients with Alzheimer’s and Parkinson’s disease. Neurobiol. Aging.

[15] Green R.C., Schneider L.S., Amato D.A., Beelen A.P., Wilcock G., Swabb E.A., Zavitz K.H. (2009). Effect of tarenflurbil on cognitive decline and activities of daily living in patients with mild Alzheimer disease: A randomized controlled trial. JAMA.

[16] Zakrzewska-Pniewska B., Gawel M., Szmidt-Salkowska E., Kepczynska K., Nojszewska M. (2012). Clinical and functional assessment of dysautonomia and its correlation in Alzheimer’s Disease. Am. J. Alzheimers Dis. Other Dement..

[17] Nedelec T., Couvy-Duchesne B., Monnet F., Daly T., Ansart M., Ganzer L., Lekens B., Epelbaum S., Dufouil C., Durrleman S. (2022). Identifying health conditions associated with Alzheimer’s disease upto 15 years before diagnosis: An agnostic study of French and Britsish health records. Lancet Digit. Health.

[18] Manocha G.D., Floden A.M., Miller N.M., Smith A.J., Nagamoto-Combs K., Saito T., Saido T.C., Combs C.K. (2019). Temporal progression of Alzheimer’s disease in brains and intestines of transgenic mice. Neurobiol. Aging.

[19] Kim J.E., Park J.J., Lee M.R., Choi J.Y., Song B.R., Park J.W., Kang M.J., Son H.J., Hong J.T., Hwang D.Y. (2019). Consipation in Tg2576 mice model Alzheimer’s disease associated with dysregulation of mechanism involving the mAChR signaling pathway and ER stress response. PLoS ONE.

[20] Stoyer N.M., Dos Santos Guilherme M., Endres K. (2020). Alzheimer’s disease in the gut-Major changes in the gut of 5xFAD model mice with ApoA1 as potnetial key player. FASEB J..

[21] Kim J., Lee J., Kim Y.-S., Park S.-H. (2022). Identifying the relationship between leisure walking and prevalence of Alzheimer’s disease and other dementias. Int. J. Environ. Res. Public Health.

[22] Buchman A.S., Boyle P.A., Yu L., Shah R.C., Wilson R.S., Bennett D.A. (2012). Total daily physical activity and the risk of AD and cognitive decline in older adults. Neurology.

[23] Lautenschlager N.T., Cox K.L., Flicker L., Foster J.K., van Bockxmeer F.M., Xiao J., Greenop K.R., Almeida O.P. (2008). Effect of physical activity on cognitive function in older adults at risk for Alzheimer’s disease: A randomized trial. JAMA.

[24] Stranahan A.M., Lee K.W., Becker K.G., ZHang Y., Maudsley S., Martin B., Cutler R.G., Mattson M.P. (2010). Hippocampal gene expression patterns underlying the enhancement of memory by running in aged mice. Neurobiol. Aging.

[25] Garcia-Mesa Y., Lopez-Ramos J.C., Gimenez-Llort L., Revilla S., Guerra R., Gruart A., Laferla F.M., Cristofol R., Delgado-Garcia J.M., Sanfeliu C. (2011). Physical exercise protects against Alzheimer’s disease in 3xTg-AD mice. J. Alzheimers Dis..

[26] Alkhouli M.F., Hung J., Squire M., Anderson M., Castro M., Babu J.R., Al-nakkash L., Broderick T.L., Plochocki J.H. (2019). Exercise and resveratrol increase fracture resistance in the 3xTg-AD mouse model of Alzheimer’s disease. BMC Complement. Altern. Med..

[27] Esfandiarei M., Hoxha B., Talley N.A., Anderson M.R., Alkhouli M.F., Squire M.A., Eckman D.M., Babu J.R., Lopaschuk G.D., Broderick T.B. (2019). Beneficial effects of resveratrol and exercise training on cardiac and aortic function and structure in the 3xTg mouse model of Alzheimer’s disease. Drug Des. Dev. Ther..

[28] Toru S., Kanouchi T., Yokota T., Yagi Y., Machida A., Kobayashi T. (2018). Utility of autonomic function tests to differentiate dementia with Lewey bodies and Parkinson disease with dementia from Alzheimer’s disease. Eur. Neurol..

[29] Billings L.M., Oddo S., Green K.N., McGaugh J.L., LaFerla F.M. (2005). Intraneural Abeta causes the onset of early Alzheimer’s disease-related conginitive deficits in transgenic mice. Neuron.

[30] Oddo S., Caccamo A., Shepherd J.D., Murphy M.P., Golde T.E., Kaved R., Metherate R., Mattson M.P., Akbari Y., LaFerla F.M. (2003). Triple-transgenic model of Alzheimer’s disease with plaques and tangles: Intracellular Abeta and synaptic dysfunction. Neuron.

[31] Broderick T.L., Rasool S., Li R., Zhang Y., Anderson M.P., Al-Nakkash L., Plochocki J.H., Geetha T., Babu J.R. (2020). Neuroprotective effects of chronic resveratrol treatment and exercise training in the 3xTg-AD mouse model of Alzheimer’s disease. Int. J. Mol. Sci..

[32] Farrer L.A., Cupples L.A., Haines J.L., Hyman B., Kukull W.A., Mayeux R., Myers R.H., Pericak-Vance M.A., Risch N., van Dujin C.M. (1997). Effects of age, sex, and ethnicity on the association between apolipprotein E genotype and Alzheimer’s Disease. JAMA.

[33] Podcasy J.L., Epperson C.N. (2016). Considering sex and gender in Alzheimer disease and other dementias. Dialogues Clin. Neurosci..

[34] Al-Nakkash L., Batia L., Bhakta M., Peterson A., Hale N., Skinner R., Sears S., Jensen J. (2011). Stimulation of murine intestinal secretion by daily genistein injections: Gender-dependent differences. Cell. Physiol. Biochem..

[35] Leung L., Kang J., Rayyan E., Bhakta A., Barrett B., Larsen D., Jelinek R., Willey J., Cochran S., Broderick T.L. (2014). Decreased basal chloride secretion and altered CFTR, villin and GLUT5 protein expression in jejunum from *ob/ob* mice. Diabetes Metab. Syndr. Obes. Targets Ther..

[36] Yamamoto Y., Okano T., Yamada H., Akashi K., Sendo S., Ueda Y., Morinobu A., Saegusa J. (2021). Soluble guanylate cyclase stimulator reduced the gastrointestinal fibrosis in bleomycin-induced mouse model of systemic sclerosis. Arthritis Res. Ther..

[37] Catmull S., Masood F., Schacht S., Dolan R., Stegman D., Leung L., Al-Nakkash L. (2016). Dietary genistein rescues reduced basal chloride secretion in diabetic jejunum via sex-dependent mechanisms. Cell. Physiol. Biochem..

[38] Lord R., Fairbourn N., Mylavarapu C., Dbeis A., Bowman T., Chandrashekar A., Banayat T., Hodges C.A., Al-Nakkash L. (2018). Consuming genistein improves survival rates in the absence of laxative in deltaF508-CF female mice. Nutrients.

[39] Knight E.M., Verkhratsky A., Luckman S.M., Allan S.M., Lawreence C.B. (2012). Hypermetabolism in a triple-transgenic mouse model of Alzheimer’s disease. Neuobiol. Aging.

[40] Stewart D.C., Berrie D., Xinyue J.L., Rickerson C., Mkoji D., Iqbal A., Tan S., Doty A.L., Glover S.C., Simmons C.S. (2018). Quantitative assessemnt of intestinal stiffness and associations with fibrosis in human inflammatory bowell disease. PLoS ONE.

[41] Alnutt A.B., Waters A.K., Kesari S., Yenugonda V.M. (2020). Physiological and pathological roles of CDK5: Potnetial directions for therapeutic targeting in neurodegenerative disease. ACS Chem. Neurosci..

[42] Patrick G.N., Zukerberg L., Nikolic M., de la Monte S., Dikkes P., Tsai L.H. (1999). Conversion of p35 to p25 deregulates Cdk5 activity and promotes neurodegeneration. Nature.

[43] Cruz J.C., Tseng H.-C., Goldman J.A., Shih H., Tsai L.-H. (2003). Aberrant Cdk5 activation by p21 triggers pathological events leading to neurodegeneration and neurofibrillary tangles. Neuron.

[44] Wilkaniec A., Czapski G.A., Adamczyk A. (2016). Cdk5 at a crossroads of protein oligomerization in neurodegenerative diseases: Facts and hypotheses. J. Neurochem..

[45] Becker C., Watson A.J., Neurath M.F. (2013). Complex roles of caspases in the pathogenesis of inflammatory bowel disease. Gastroenterology.

[46] Lange-Asschenfeldt C., Kojda G. (2008). Alzheimer’s disease, cerebrovascular dysfunction and the benefits of exercise: From vessels to neurons. Exp. Gerontol..

[47] Vidoni E.D., Honea R.A., Billinger S.A., Swerdlow R.H., Burns J. (2012). Cardiorespiratory fitness is associated with atrrophy in Alzheimer’s and aging over 2 years. Neurobiol. Aging.

[48] Santana-Sosa E., Barriopedro M.I., Lopez-Mojares L.M., Perez M., Lucia A. (2008). Exercise training is beneficial for Alzheimer’s patients. Int. J. Sports Med..

[49] Puente-Gonzalez A.S., Sanchez-Shanchez M.C., Fernandez-Rodriguez E.J., Hernandez-Xumet J.E., Babero-Iglesias F.J., Mendez-Sanchez R. (2021). Effects of 6-month multimodal physical exercise program on bone mineral density, fall risk, balance, and gait in patients with Alzheimer’s disease: A controlled clinical trial. Brain Sci..

[50] Rajesh Y., Kannegarti T.-D. (2022). Innate immune cell death in neuroinflammation and Alzheimer’s disease. Cells.

[51] Bertoni S., Ballabeni V., Flammini L., Gobbetti T., Impicciatore M., Barocelli E. (2008). Intestinal chronic obstruction affects motor responsivemenss of rat hypertrophic longitudinal and circular muscles. Neurogastroenterol. Motil..

[52] da Cunha Araujo L.C., de Souza L.L.L., Vasconcelos L.H.C., de Fritos Brito A., Queiroga F.R., Silva A.S., da Silva P.M., de Andrade Cavalcante F., da Silva B.A. (2015). Chronic aerobic swimming exercise promotes functional and morphological changes in rat ileum. Biosci. Rep..

[53] Aczel D., Gyorgy B., Bakonyi P., Bukhari R., Pinho R., Boldogh I., Yaodong G., Radak Z. (2022). The systemic effects of exercise on the systemic effects of exercise. Antioxidants.

[54] Ahmed Z., Cooper J., Murray T.K., Garn K., McNaughton E., Clarke H., Parhizkar S., Ward M.A., Cavallini A., Jackson S. (2014). A novel in vivo model of tau propagation with rapid and progressive neurofilbrillary tangle pathology: The pattern of spread is determined by connectivity, not proximity. Acta Neuropathol..

[55] Han X., Tang S., Dong L., Song L., Dong Y., Wang Y., Du Y. (2017). Loss of nitrergic and cholinergic meurons in the enteric nervous system of APP/PS1 transgenic mouse model. Neurosci. Lett..

[56] Puig K.L., Lutz B.M., Urqhart S.A., Rebel A.A., Zhou X., Manocha G.D., Sens M., Tuteja A.K., Foster N.L., Combs C.K. (2015). Overespression of mutant amyloid-B-protein precurser and presnillin 1 modulates enteric nervous system. J. Alzheimers Dis..

[57] Sun Y., Sommerville N.R., Liu J.Y.H., Ngan M.P., Poon D., Ponomarev E.D., Lu Z., Kung J.S.C., Rudd J.A. (2020). Intra-gastrointestinal amyloid-B1-42 oligomers preturb enteric function and induce Alzheimer’s disease pathology. J. Physiol..

[58] Van Ginneken C., Schafer K.-H., Van Dam D., Huygelen V., De Deyn P.P. (2011). Morphological changes in the enteric nervous system of agining and APP23 transgenic mice. Brain Res..

[59] Lee M.-H., Amin N.D., Venkatesan A., Wang T., Tyagi R., Pant H.C., Nath A. (2013). Impaired neurogenesis and neurite outgrowth in an HIV-gp120 transgenic model is reversed by exercise via BDNF production and Cdk5 regulation. J. Neurovirol..

